# High mitochondrial mass identifies a sub-population of stem-like cancer cells that are chemo-resistant

**DOI:** 10.18632/oncotarget.5401

**Published:** 2015-10-03

**Authors:** Gillian Farnie, Federica Sotgia, Michael P. Lisanti

**Affiliations:** ^1^ Cancer Stem Cell Research, Institute of Cancer Sciences, University of Manchester, UK; ^2^ The Breast Cancer Now Research Unit, Institute of Cancer Sciences, University of Manchester, UK; ^3^ The Manchester Centre for Cellular Metabolism (MCCM), Institute of Cancer Sciences, University of Manchester, UK

**Keywords:** mitochondria, MitoTracker, cancer stem cells, tumor metabolism

## Abstract

Chemo-resistance is a clinical barrier to more effective anti-cancer therapy. In this context, cancer stem-like cells (CSCs) are thought to be chemo-resistant, resulting in tumor recurrence and distant metastasis. Our hypothesis is that chemo-resistance in CSCs is driven, in part, by enhanced mitochondrial function. Here, we used breast cell lines and metastatic breast cancer patient samples to begin to dissect the role of mitochondrial metabolism in conferring the CSC phenotype. More specifically, we employed fluorescent staining with MitoTracker (MT) to metabolically fractionate these cell lines into mito-high and mito-low sub-populations, by flow-cytometry. Interestingly, cells with high mitochondrial mass (mito-high) were specifically enriched in a number of known CSC markers, such as aldehyde dehydrogenase (ALDH) activity, and they were ESA+/CD24-/low and formed mammospheres with higher efficiency. Large cell size is another independent characteristic of the stem cell phenotype; here, we observed *a* >2-fold increase in mitochondrial mass in large cells (>12-μm), relative to the smaller cell population (4–8-μm). Moreover, the mito-high cell population showed a 2.4-fold enrichment in tumor-initiating cell activity, based on limiting dilution assays in murine xenografts. Importantly, primary human breast CSCs isolated from patients with metastatic breast cancer or a patient derived xenograft (PDX) also showed the co-enrichment of ALDH activity and mitochondrial mass. Most significantly, our investigations demonstrated that mito-high cells were resistant to paclitaxel, resulting in little or no DNA damage, as measured using the comet assay. In summary, increased mitochondrial mass in a sub-population of breast cancer cells confers a stem-like phenotype and chemo-resistance. As such, our current findings have important clinical implications for over-coming drug resistance, by therapeutically targeting the mito-high CSC population.

## INTRODUCTION

Cancer stem-like cells (CSCs) or tumor-initiating cells (TICs) are thought to be responsible for driving patient relapse (tumor recurrence and metastasis), as a consequence of their chemo-resistance and/or radio-resistance [1–6]. However, the cellular mechanism(s) driving this drug-resistant phenotype remain largely unknown. Elucidation of the mechanisms underpinning drug-resistance would have broad clinical implications, for the prevention of treatment failure in a variety of different cancer types.

One hypothesis is that mitochondria are involved in conferring drug-resistance, as they control both i) energy metabolism and ii) susceptibility towards apoptotic cell death. Previous studies have shown that catabolic cancer-associated fibroblasts (CAFs) produce mitochondrial fuels (such as L-lactate, ketone bodies and L-glutamine), which are sufficient to confer a drug-resistant phenotype in MCF7 breast cancer cells in culture [7, 8]. In this context, treatment with metformin (a mitochondrial inhibitor of Complex I) was sufficient to revert this drug-resistance phenotype. Similar results were also obtained in a variety of distinct patient cohorts followed at the MD Anderson and Dana-Farber Cancer Centers, showing that increased mitochondrial integrity (in stained tumor-tissue sections) was associated with drug-resistance and treatment failure in a number of different cancer types, including multiple myeloma, acute myelogenous and lymphoblastic leukemia, as well as ovarian cancers [9, 10].

Our group has previously shown that breast CSCs are chemo- and radio-resistant and that stem cell regulatory pathways, such as Notch, Wnt, FAK and CXCR1/2, may play a role in their maintenance and resistance mechanisms [11–17]. However, there is a limited understanding of the metabolic activities within CSC-enriched and CSC-depleted populations, although mechanistic differences are undoubtedly present [18–20]. Metabolic investigations in glioma stem cells (GSCs) showed that radio-resistant GSCs were less glycolytic, with a higher mitochondrial reserve capacity [18], indicating that specific inhibition of OXPHOS may target the GSCs. Similarly CSCs isolated from ovarian cancer patients also showed evidence of a metabolic profile dominated by OXPHOS, even in the absence of glucose. The authors suggest that these findings could be instrumental in helping CSCs to escape damage in hypo-oxygenated tumor areas [21].

To determine if breast CSC characteristics were associated with a specific metabolic activity, we used MitoTracker (MT) as an investigational tool to live-stain and metabolically fractionate cancer cell lines and patient samples into mito-high and mito-low cell populations. Our study demonstrates that cells with high mitochondrial mass showed many of the characteristics of CSCs, including increased ALDH activity, mammosphere formation activity and tumor initiation *in vivo*. In addition, we show increased survival and decreased DNA damage within the mito-high cells after paclitaxel treatment, suggesting that mitochondrial mass confers a chemo-resistance phenotype, as predicted.

Thus, our current findings provide a new mitochondrially-based model for understanding the relationship between CSCs and chemo-resistance. As this approach is technically simple, it could be used to rapidly isolate chemo-resistant CSC populations from possibly any tumor type, facilitating the development of new classes of drugs to target chemo-resistance.

## RESULTS

### High mitochondrial mass directly correlates with ALDH activity, the ESA+CD24-/low CSC population and larger cell size

Studies show CSC and non-CSC populations may use different metabolic pathways and this may contribute to their increased survival and resistance to chemotherapy [18, 22, 23]. To determine if mitochondrial mass is increased within the CSC population, we fluorescently-labeled mitochondria in MCF7 and MDA MB 231 breast cancer cells with MitoTracker Deep-Red. These cells were co-stained with ALDEFLUOR or CSC cell surface markers ESA/CD24, to identify the CSC population. Both ALDH+ and ESA+/CD24− cell populations have been shown to be enriched in mammosphere- and tumor-initiating cells, key characteristics of CSCs [2, 24–28].

**Figure 1:**
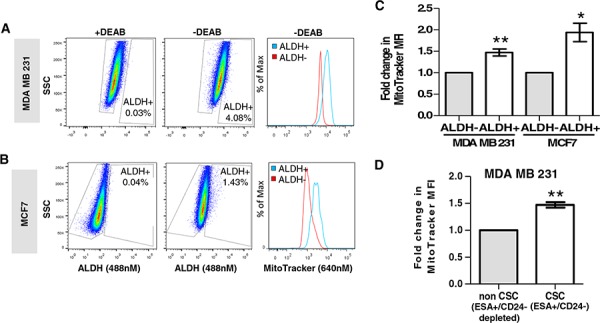
Mitochondrial mass directly correlates with ALDH activity and the ESA+CD24-/low CSC population Representative dot plots of ALDH activity in MDA MB 231 **A.** and MCF7 **B.** cells, showing ALDH+ and ALDH− cells in the absence of DEAB. Histograms represent typical staining intensity of MitoTracker in ALDH+ and ALDH− populations in both cell lines. **C.** Graph showing fold change in mean fluorescence intensity (MFI) of MitoTracker (Deep Red; 640 nM), within ALDH+ and ALDH− populations of MCF7 and MDA MB 231 cell lines (*n* = 4 independent experiments). **D.** Graph showing fold change in mean fluorescence intensity of MitoTracker (Deep Red; 640 nM) within the ESA+CD24− (cancer stem-like cell, CSC) population and ESA+/CD24− depleted (non-CSC) populations of MDA MB 231 cells (*n* = 4 independent experiments). Bar graphs are shown as the mean ± SEM, *t*-test, two-tailed test, *P ≤ 0.05, **P ≤ 0.01.

Figure 1A, 1B, 1C shows that MitoTracker staining is specifically enriched in the ALDH+ cell population of MCF7 and MDA MB 231 cells (*P* < 0.05). A similar fold increase in MitoTracker mean fluorescence intensity was also observed in the ESA+CD24-/low CSC population of the MDA MB 231 cell line (Figure 1D, *P* < 0.01). These findings suggest that CSCs contain a higher mitochondrial mass than the non-CSC population.

**Figure 2:**
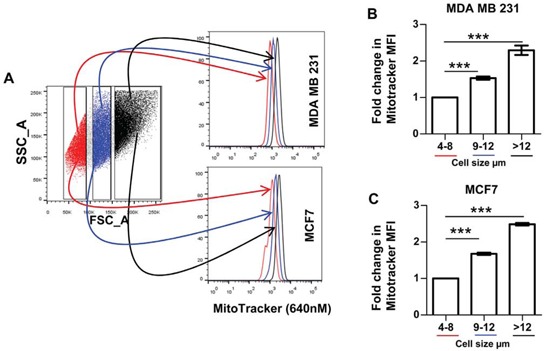
Mitochondrial mass directly correlates with the enriched breast CSC population, identified using large cell size **A.** Typical dot plot showing side scatter (SSC) and forward scatter (FSC) of live breast cancer cells, gates represent cell size (RED- 4–8 μm; BLUE- 9–12 μm; BLACK > 12 μm; [29]). Histograms show MitoTracker mean fluorescence intensity within the 3 cell size groups of MDA MB 231 and MCF7 cells. Graphs showing fold change in the mean fluorescence intensity (MFI) of MDA MB 231 **B.** and MCF7s **C.** within the 9–12 μm and > 12 μm cell size compared to the smallest cells (4–8 μm), *n* = 3 independent experiments, 2 technical replicates. Bar graphs are shown as the mean ± SEM, *t*-test, two-tailed test, ***P < 0.001.

As an alternative approach to enrich CSCs, we used cell size. Previous studies have shown that cells with mammary stem cell activity tend to be larger than 10 μm [29]. As a consequence, we used forward scatter (FSC) to isolate three different cell populations, based solely on size: 4–8 μm, 9–12 μm and >12 μm (Figure 2A). Quantitative analysis of MitoTracker staining demonstrated that larger cells were associated with significantly higher mitochondrial mass, up to 2.5-fold, consistent with an anabolic CSC phenotype (Figure 2B and 2C, *P* < 0.001).

These data indicate that high mitochondrial mass, as determined by MitoTracker staining, is associated with breast CSC populations enriched via three independent CSC markers, namely ALDH activity, ESA/CD24 cell surface levels or cell size.

### High mitochondrial mass directly correlates with ALDH activity in primary breast cancer cells isolated from metastatic disease sites or a patient derived xenograft (PDX)

To validate the possible *in vivo* relevance of our above findings, we next examined mitochondrial mass in primary CSC populations from metastatic breast cancer patients. For this purpose, we co-labeled breast cancer cells isolated directly from pleural effusions or ascites fluids (*n* = 4) with ALDEFLUOR and MitoTracker.

**Figure 3:**
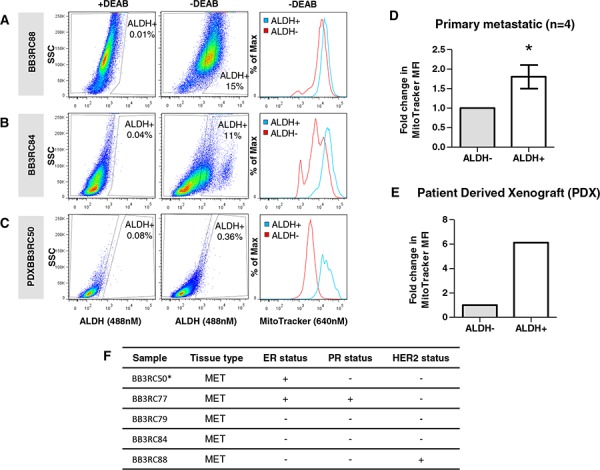
Mitochondrial mass correlates with ALDH activity in primary breast cancer cells isolated from metastatic breast cancer samples and a patient derived xenograft Representative dot plots of ALDH activity in primary metastatic breast cancer samples BB3RC88 **A.** BB3RC84 **B.** and patient derived xenograft sample (BB3RC50) **C.** cells showing ALDH+ and ALDH− cells in the absence of DEAB. Histograms represent typical staining intensity of MitoTracker (MT) in ALDH+ and ALDH− populations. **D.** Graph showing fold change in mean fluorescence intensity (MFI) of MitoTracker (Deep Red; 640 nM) within ALDH+ and ALDH− populations of primary metastatic breast cancer samples (*n* = 4), BB3RC77, BB3RC79, BB3RC88, BB3RC84. **E.** Graph showing fold change in mean fluorescence intensity (MFI) of MitoTracker (Deep Red; 640 nM) within ALDH+ and ALDH− populations of a patient derived xenograft sample (BB3RC50). **F.** Table showing the original (invasive) breast cancer characteristics of the metastatic (MET) breast cancer samples. Estrogen receptor (ER) and progesterone receptor (PR) status, + = > 10% positive; HER2 status, + = 3+ or 2+ & amplified; BB3RC50* denotes the PDX sample. Bar graphs are shown as the mean ± SEM, *t*-test, two-tailed test, *P ≤ 0.05.

Figure 3A, 3B, and 3D supports our breast cancer cell line data, showing that ALDH+ primary metastatic breast CSCs have significantly higher mitochondrial mass than the ALDH− cells (*P* < 0.05). Notably, although these findings are of a low sample size, our results appear to be independent of estrogen receptor (ER), progesterone (PR) and HER2 status (Figure 3F). In addition, we also show similar results within the ALDH+ population of human breast cancer cells isolated from a patient derived xenograft (BB3RC50*) (Figure 3C and 3E). These data suggest that high mitochondrial mass is associated with CSC populations from freshly isolated metastatic breast cancer cells.

### High mitochondrial mass enriches for mammosphere-forming activity *in vitro* and tumor-initiating activity *in vivo*

**Figure 4:**
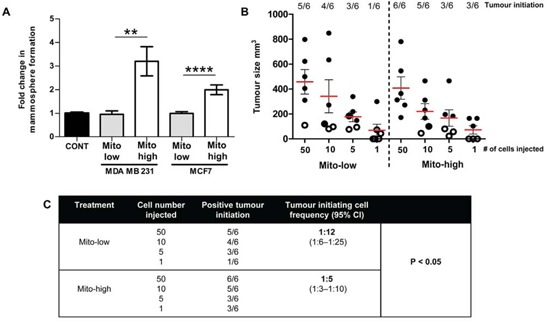
High mitochondrial mass is specifically associated with mammosphere formation and tumor-initiating activity MDA MB 231 and MCF7 cells were subjected to flow-cytometry to isolate different populations of MitoTracker (MT) stained cells: mito-low = lowest 5% of MitoTracker stained cells; mito-high = highest 5% of MitoTracker stained cells. Mito-high and low populations were then seeded into mammosphere cultures **A.** or injected by limiting dilution into NOD scid gamma (NSG) mice **B.** (A) Graph showing fold change in mammosphere formation in mito-low and mito-high populations of MDA MB 231 and MCF7 cells, *n* = 4 independent experiments, ≥3 technical replicates. (B) Graph showing tumor size (mm^3^), 9 weeks after cell injections (sub-cutaneous), with 1, 5, 10 and 50 cells per injection of mito-low and mito-high MDA MB 231 cell populations. Each dot represents a tumor, hollow dots <100 μm = no tumor initiation. **C.** Extreme limiting dilution analysis (ELDA) was used to calculate the tumor initiation cell frequency within the mito-low and mito-high MDA MB 231 cell populations. Graphs are shown as the mean ± SEM, *t*-test, two-tailed test, **P ≤ 0.01, **** ≤ 0.0001.

Although we correlated high mitochondrial mass with a number of CSC markers, it was important to validate our findings with more functional parameters, such as mammosphere-forming activity and tumor initiation, that have been traditionally associated with “stemness” [30–32]. MCF7 and MDA MD 231 cells were sorted into mito-high (top 5% of cells with high MitoTracker staining) and mito-low (bottom 5% of cells with low MitoTracker staining) cell populations using flow-cytometry. The mito-high and mito-low cell populations were then seeded under non-adherent culture conditions and the number of mammospheres >60 μm were counted after 5 days. Both MCF7 and MDA MB 231 cells showed a significant 2- to -3 fold increase in mammosphere forming efficiency within the mito-high population, compared to mito-low cells (Figure 4A, *P* < 0.01).

The most stringent test of CSC activity is the capacity for tumor initiation [31, 32]. Serial dilutions of MDA MB 231 mito-high and mito-low cells (1, 5, 10, and 50 cells) were injected into NOD scid gamma (NSG) mice, and tumor initiation and growth were measured over 9 weeks. Our results show high mitochondrial mass was not associated with increased tumor growth/size (Figure 4B). However, using extreme limiting dilution analysis (ELDA), we demonstrate a 2.4-fold increase in tumor initiation cell frequency within the mito-high population of MDA MB 231 cells, when compared to mito-low (Figure 4C, *P* < 0.05). Although high mitochondrial mass was not associated with increased tumor size (Figure 4B), these data show that high mitochondrial mass is specifically associated with two key functional CSC characteristics, *in vitro* mammosphere formation and tumor-initiating cell frequency *in vivo*.

### CSCs with high mitochondrial mass preferentially survive paclitaxel treatment and show reduced DNA strand breaks, conferring chemo-resistance

Another key CSC characteristic is resistance to radio- and chemo-therapy [2, 3, 17]. In order to correlate chemo-resistance with mitochondrial mass, MCF7 and MDA MB 231 cells were first separated into mito-high and mito-low cell fractions by flow-cytometry, using MitoTracker. The two cell populations were then seeded into mammosphere culture in the presence or absence of paclitaxel (0.1–0.5 μM) to assess their survival characteristics and susceptibility towards DNA damage.

**Figure 5:**
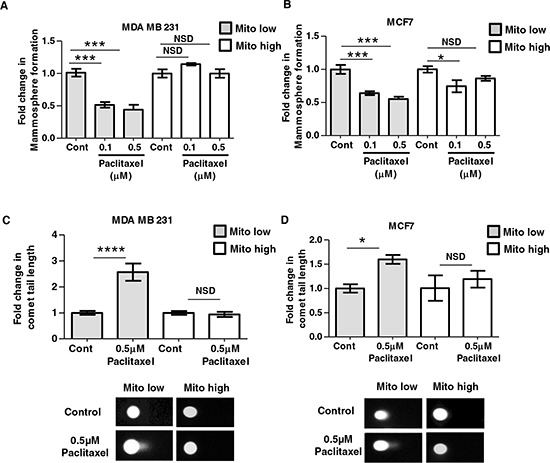
CSCs with high mitochondrial mass preferentially survive paclitaxel treatment and show reduced DNA strand breaks MDA MB 231 and MCF7 cells were subjected to flow-cytometry to isolate different populations of MitoTracker stained cells: mito-low = lowest 5% of MitoTracker stained cells; mito-high = highest 5% of MitoTracker stained cells. Mito-high and low populations were then seeded into mammosphere culture in the presence or absence of paclitaxel (A–B) or treated with paclitaxel or control (DMSO 1:10,000) for 24 h before DNA damage analysis via a comet assay (C–D). **A–B.** Graphs show fold change in mammosphere formation in mito-low and mito-high cells (MDA MB 231 and MCF7), after treatment with 0.1 μM and 0.5 μM paclitaxel compared to vehicle alone controls, *n* = 3 independent experiments, ≥3 technical replicates. **C–D.** Graph shows fold change in comet tail length in mito-low and mito-high cell populations (MDA MB 231 and MCF7), after 24 h of 0.5 μM paclitaxel treatment, the images show representative comet tails under all conditions. Bar graphs are shown as the mean ± SEM, (ANOVA) test with post-hoc dunetts multiple comparisons (A–B), *t*-test, two-tailed test (C–D), **P ≤ 0.01, **** ≤ 0.0001.

Figure 5A and 5B shows that cells with high mitochondrial mass are resistant to paclitaxel, demonstrating little or no decrease in mammosphere formation, compared to vehicle alone controls. In contrast, cells with low mitochondrial mass show significant reductions in mammosphere formation, up to 2-fold, at the same concentrations (*P* < 0.001). Thus, high mitochondrial mass directly correlates with resistance to paclitaxel in CSCs.

To better mechanistically understand this chemo-resistance phenotype, we used the comet assay to functionally measure the levels of DNA strand breaks after 24 h of paclitaxel treatment [33]. Remarkably, Figure 5C and 5D shows that cells with high mitochondrial mass have significantly reduced DNA damage, whereas cells with low mitochondrial mass are more chemo-sensitive, with increased comet tail length after paclitaxel treatment. These data indicate that high mitochondrial mass allows CSCs to survive chemotherapy, by affording protection or increased repair against DNA damage.

## DISCUSSION

Drug resistance is a rate-limiting barrier to successful cancer therapy. Tumor-initiating cells and/or CSCs share a common chemo-resistant phenotype, which ultimately drives tumor recurrence and metastatic disease, resulting in patient relapse [1–6]. In this report, we show that high mitochondrial mass is associated with CSC markers, functional CSC characteristics (mammosphere and tumor initiation) and chemo-resistance. Our findings highlight the need to further understand and target the metabolic pathways in CSCs, to improve clinical outcome in breast cancer patients.

ALDEFLUOR is an investigational tool, which is routinely used to measure ALDH activity in cell lines and primary tumor samples, enriching for CSC activity via flow-cytometry [27, 28]. However, the ALDH gene family is quite large and it still remains controversial which ALDH isoforms contribute towards ALDH activity that is associated with CSC activity [34–37]. As our current report shows that ALDH activity in CSCs is also associated with high levels of mitochondrial mass, in cell lines and patient samples, it is quite possible that this stem-cell associated ALDH activity may also be derived from mitochondria. In fact, at least 7 members of the ALDH family of genes are known to be targeted to mitochondria and are *bonafide* resident mitochondrial proteins, including ALDH2, ALDH1L2, ALDH4A1, ALDH5A1, ALDH6A1, ALDH7A1 and ALDH18A1. Therefore, this new mitochondrial connection could functionally explain the relationship between ALDH activity and stemness in cancer cells. In support of this idea, it was recently shown that the drug-resistant ALDH-high cell sub-population has significantly increased mitochondrial respiration, leading to increased levels of oxidative stress [38].

We also showed that other CSC markers (ESA/CD24), and large cell size were associated with high mitochondrial mass. This supports other published studies where breast CSCs have been shown to have a higher maximum mitochondrial capacity and mitochondrial proton leak, compared to their differentiated non-CSC progeny [22]. Moreover, we demonstrated that cells with high mitochondrial mass were also functionally more stem-like, showing significant increases in i) mammosphere-forming efficiency and ii) tumor-initiating cell activity *in vivo*. The mechanism behind the increased mitochondrial mass in our study remains unknown. However, CSC populations are known to have a distinct genomic and proteomic expression profile [39], which can contribute to the stem cell characteristics of CSCs. This differential expression may also influence mitochondrial biogenesis, for example high levels of mTORC1 and its activity are seen in CSC populations of pancreatic and breast cancer cells [40, 41]. Studies have shown mitochondrial activity and biogenesis are controlled by mTORC1 by promotion of the translation of mitochondria-related mRNAs, via inhibition of the eukaryotic translation initiation factor 4E (eIF4E)-binding proteins [42], suggesting the high activity of mTORC1 in CSCs may increase mitochondrial biogenesis. Similarly, the transcription coactivator peroxisome proliferator-activated receptor gamma, coactivator 1 alpha (PGC-1α), a known mediator of mitochondrial biogenesis, has been shown to be overexpressed in circulating tumor cells (with CSC characteristics) from the MMTV-PyMT spontaneous mouse breast cancer model [43]. The over-expression of oncogenes is also linked to increased mitochonidrial mass. Increased expression of Myc maintained the stemness of brain stem cells and glioma CSCs [44, 45] but also stimulated nuclear-encoded mitochondrial genes and mitochondrial biogenesis [46]. These studies suggest the differential expression of proteins and genes within the CSC population may preferentially favor increased mitochondrial biogenesis, compared to the non-CSC population.

The inherent expression of genes that increase mitochondrial biogenesis may, in turn, aid the maintenance of stem cell characteristics, as mitochondrial function has been shown to be important for the self renewal and differentiation capacity of stem cells. Although mitochondrial mass in embryonic stem cells is low, studies in normal adult stem cells and CSCs demonstrate the levels of mitochondrial mass are tissue-specific [47]. In glioblastoma, the depletion of mitochondrial DNA, after 2′-3′-dideoxycytidine treatment, inhibited tumor initiation [48], whereas reduced mitochondrial mass identified lung cancer stem cells [49]. A recent paper also highlighted the asymmetric apportioning of old/aged mitochondria, where the retention of young mitochondria during asymmetric self-renewal was vital to maintaining the stem cell characteristics of normal mammary epithelial cells [50]. Our data suggests that increased mitochondrial mass may increase tumor initiation via increased self renewal. Further investigations on the tumor initiation capacity of breast CSCs after the depletion or inactivation of mitochondria are warranted to validate this hypothesis.

Although high mitochondrial mass increased tumor initiation, especially at low cell numbers, it did not significantly affect overall tumor growth/size. These findings may simply reflect the recent observation that tumor cells have the capacity to “steal” mitochondrial DNA (mt-DNA) from host stromal cells (such as mesenchymal stem cells or endothelial cells), to boost their metabolic capacity towards OXPHOS, depending on the length of time they spend in the host [51]. Interestingly, it has also been reported that the transfer of stromal cell mitochondria to cancer cells increases their respiratory capacity and chemo-resistance [52].

Our findings also demonstrated that cells with high mitochondrial mass were resistant to chemotherapy, effectively protecting, delaying or avoiding DNA strand breaks after 24 h of paclitaxel treatment. Other studies show that inhibiting ALDH activity, which is increased within the mito-high population, can increase chemo-sensitivity [53, 54], although the specific mechanism is currently unknown. Evidence also shows that chemo-resistance solid cancers could be driven by numerous metabolic mechanisms, including reduced reactive oxygen species [55] and increased mitochondrial coupling [56]. Similarly, a recent study has identified that SIRT4, a mitochondria-localized sirtuin, regulates the metabolic responses to DNA damage by repressing mitochondrial glutamine metabolism. This contributes to the control of cell cycle progression and the maintenance of genomic integrity in response to DNA damage [57]. Differential metabolic control of the cell cycle may therefore play a role in reducing DNA damage in our study, as paclitaxel requires cell division to induce microtubule damage. Further exploration to elucidate the exact mechanisms is warranted.

CSCs are known to have increased DNA damage responses and repair mechanisms, characterized by the expression of high levels of DNA repair proteins, e.g. Chk1 and Rad51 [58, 59]. Since our mito-high cell population is enriched in established CSC markers, it is reasonable to presume the reduced DNA damage may be facilitated in part via non-metabolic mechanisms. However, recent studies have described signaling linking the metabolite fumarate to the promotion of DNA repair. Increased fumarate inhibited KDM2B histone demethylase activity, which subsequently increased accumulation of DNA-PK at double strand break regions, driving non-homologous end-joining DNA repair and cell survival [60, 61]. Research to date has only just started to reveal the complex signaling cross-talk linking metabolic processes with the DNA damage response and other biological functions. Further investigations will aid our understanding and deliver new targets to improve cancer treatment.

Interestingly, most markers of stemness that are routinely used to enrich for CSC activity (CD24, ESA, CD133, KRT19) are not generally thought of as therapeutically druggable targets. In contrast, the discovery of mitochondrial mass as a biomarker of CSC activity, as detected using the MitoTracker probe, suggests that we could therapeutically target mitochondria to more effectively eradicate the CSC population. There are many potential targets within mitochondria that could be inhibited to improve cancer treatment. These include targeting mitochondrial control of apoptosis with inhibitors the BCL-2 family [62] or HSP90, which has been shown to be specifically expressed in cancer cell mitochondria, but not their normal counterparts [63, 64]. Specific targeting of upregulated metabolic pathways, such as fatty acid synthesis, glycolysis and OXPHOS have also been shown to have anti-cancer effects (reviewed in Ref [65]). As discussed above, mitochondrial biogenesis can be induced by the hyper-activation of numerous signaling pathways and oncogenes, such as mTOR and Myc, which can be inhibited using rapacycin analogues e.g. everolimus [66] or DCR-MYC, a novel siRNA-based therapeutic, designed to silence the MYC oncogene which is currently in Phase I clinical trials [67].

The “Endo-symbiotic Theory of Mitochondrial Evolution” suggests that mitochondria originally evolved from engulfed aerobic bacteria, over millions of years of adaptation [68, 69]. As such, antibiotics target mitochondria, and therefore this side effect could be exploited to target the increased mitochondrial mass within CSCs. Interestingly, several FDA-approved drugs/antibiotics that target mitochondria have already been shown to eradicate CSC activity, such as OXPHOS inhibitors (metformin and pyrvinium pamoate) [70–73], a potassium ionophore (salinomycin) [74, 75] and known inhibitors of mitochondrial biogenesis and mitochondrial translation (the erythromycins, the tetracyclines and the glycylcyclines) [76, 77]. In fact, doxycycline and azithromycin have already shown significant efficacy in treatment-resistant cancer patients, with MALT lymphoma and non-small cell lung tumors, respectively [78–80].

**Figure 6:**
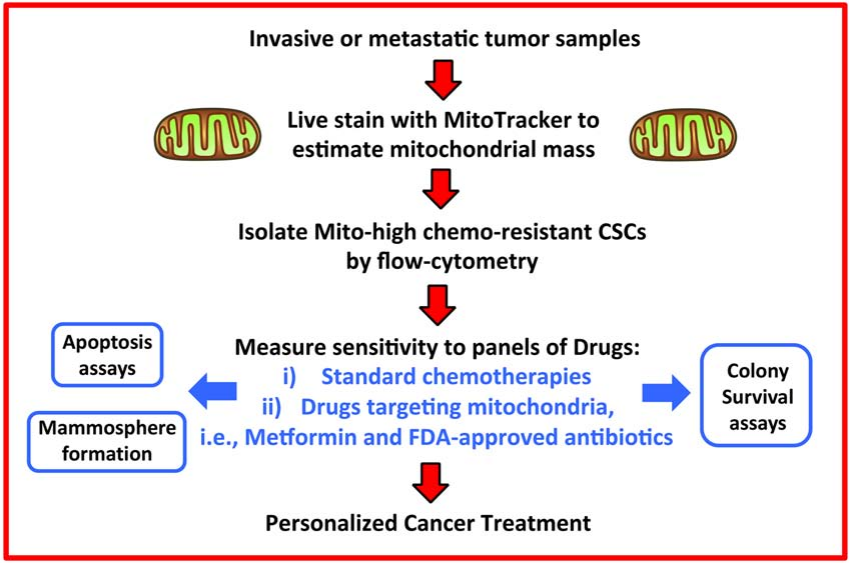
Therapeutic targeting of chemo-resistant CSCs: A new systematic approach Here, we propose a new clinical strategy for over-coming drug resistance. We suggest that primary or metastatic clinical samples could be used to purify chemo-resistant “mito-high” CSCs by flow-cytometry, after live-staining with MitoTracker, to estimate mitochondrial mass. Then, these chemo-resistant CSCs would be subjected to phenotypic drug screening, with well-defined panels of i) conventional chemotherapies and/or ii) mitochondrially-targeted FDA-approved drugs (e.g., metformin and antibiotics). This would allow us to achieve the goals of personalized cancer treatment, by establishing the chemo-sensitivity profiles of mito-high CSCs in individual patients, leading to the prevention of tumor recurrence and metastasis, in multiple cancer types. Chemo-resistant “mito-high” CSCs could also be expanded by using 3D-spheroid cultures, possibly for biobanking and drug screening.

The discovery that MitoTracker can identify a live population of cancer cells (mito-high) that are enriched for chemo-resistant cells may have a potential role in personalized medicine. For example the isolation of mito-high cells from patient samples could be used to screen for sensitivity to current or new therapies, by measuring cell death, mammosphere formation, or by employing a simple colony survival assay (Figure 6). Mito-high cells could also be isolated for genomic and proteomic analyses. Detailed analysis of the transcriptome and the proteome of mito-high cells, as compared to the mito-low cell populations, could reveal specific pathways that mito-high cells depend on for survival and maintenance. These specific pathways could then be targeted, to eliminate this chemo-resistant cell population. With further development, our new findings could have important clinical relevance for targeting CSCs, by improving sensitivity to chemotherapy and aiding in the discovery of new therapeutics that inhibit mitochondrial biogenesis in CSCs.

**Figure 7:**
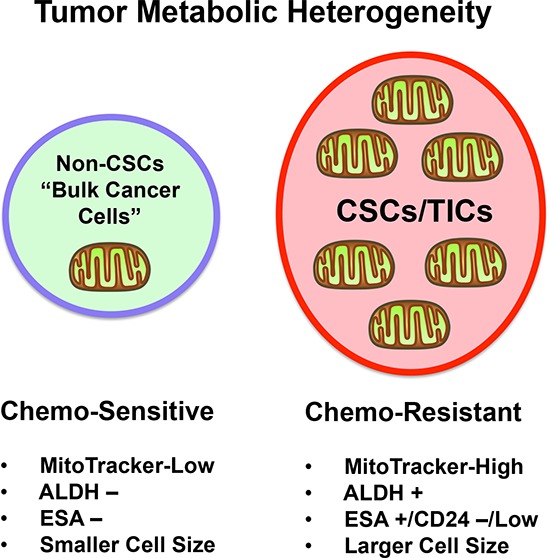
Understanding the relationship between mitochondrial mass, “stemness” and chemo-resistance in cancer cells Our report suggests that high mitochondrial mass, quantified using live MitoTracker staining, is associated with a number of stem cell characteristics and biological functions including CSC markers ALDH+, ESA/CD24, large cell size, mammosphere formation *in vitro*, as well as tumor-initiating activity *in vivo*. Moreover, cells with high mitochondrial mass were preferentially resistant to paclitaxel during mammosphere formation and the comet assay (a marker DNA damage), when compared to low mitochondrial mass cells. Thus, we conclude that high mitochondrial mass can be used as a biomarker to isolate a sub-population of stem-like cancer cells that are chemo-resistant. This has important implications for better appreciating the role of tumor metabolic heterogeneity in driving chemo-resistance and treatment failure, via recurrence and metastasis.

In summary, metabolic fractionation of cancer cell lines and primary metastatic breast cancer samples, identified a stem-like, mitochondrial-rich sub-population of tumor cells (Figure 7). CSCs with high mitochondrial mass showed an increased capacity for mammosphere formation, tumor initiation and were resistant to DNA-damage induced by paclitaxel. Thus, these results provide a new mitochondrially-based model for understanding the molecular relationship between CSCs and chemo-resistance.

## MATERIALS AND METHODS

### Cell lines and media

MCF7 and MDA MB 231 cell lines were purchased from ATCC and maintained in monolayer media—DMEM, supplemented with 10% (v/v) fetal calf serum and 200 mM L-glutamine (Sigma). Mammosphere media consisted of DMEM/F12, phenol-red-free, supplemented with 1 × B27 without vitamin A (Gibco), plus 20 ng/ml human EGF (Milteny Biotech).

### Human tumor tissues

Metastatic breast cancer sample were obtained from pleural effusions or ascites. Approval for these samples was granted by the Christie Medical Research and Biobank Ethics Committees. All patients provided written informed consent.

### Flow-cytometry analysis and sorting

#### ALDEFLUOR activity

5 × 10^5^ cells were re-suspended in 1 ml Assay buffer and 5 μl ALDEFLUOR reagent (Stem Cell Technologies) was added and mixed well. 0.5 ml of this cell suspension was transferred into a new tube with 5 μl of DEAB regent (Stem Cell Technologies). Tubes were incubated for 30 mins at 37°C before centrifugation and re-suspension in Assay Buffer (0.5 ml) for FACS analysis (BD, LSR Fortessa). When combined with MitoTracker Deep-Red (Molecular Probes, 0.125 μl/ml), both were added before 30 min incubation. Note the PDX model BB6RC50 was also co-stained with anti-mouse MHC Class I (H-2Kd) antibody conjugated with Pacific Blue (BioLegend, 116616), to exclude mouse cells in the ALDH analysis.

#### Mitotracker staining

Exponentially dividing MCF7 and MDA MB 231 cells were trypsinized and re-suspended into a 1 × 10^6^ cell/ml solution in PBS. 0.125 μl/ml MitoTracker Deep-Red (Molecular Probes) was added for 30 mins at 37°C before centrifugation and re-suspension in PBS for FACS analysis (BD, LSR Fortessa) or Sorting (BD, Aria III).

#### CSC cell surface markers

Exponentially dividing MDA MB 231 cells were trypsinized and re-suspended into a 1 × 10^6^ cell/100 μl PBS. MitoTracker (0.125 μl/ml) was added to cell suspension for 30 mins at 37°C before centrifugation and re-suspension in 100 μl PBS and the addition of 10 μl ESA-FITC (DAKO, BerEP4), 10 μl CD24-PE (Pharmingen) for 10 mins at 4°C before centrifugation and re-suspension in PBS for FACS analysis (BD, LSR Fortessa. All tubes were incubated with 1 μl/ml live/dead fixable violet cell stain (Molecular Probes) to exclude dead cells from FACS analysis and sorting.

### Comet assay

Alkaline comet assays were modified from [33]. MDA MB 231 and MCF7 cells were FACS sorted for the 5% highest and lowest MitoTracker expressing cells. Cells were seeded into 24 well plates (2 × 10^4^ cells) and treated with 0.1 μM paclitaxel for 24 h. Cell were them trypsinized and resuspended into 250 μl cell media; 750 μl of low melting point agarose (1% w/v in PBS, at 40^°^C) was added to the cell suspensions before pipetting on to a pre-agarose coated glass slide and a glass cover slip was placed on top. After 15 min at 4°C, slides were placed in lysis buffer (2.5 M NaCl, 1 mM EDTA, 10 mM Tris, 10% DMSO, 1% Triton X-100, pH 10) and left at 4°C overnight. Slides were placed in alkaline solution (0.3 M NaOH, 1 mM EDTA, pH13) for 50 mins at 4°C and electrophoresed at 23 V for 50 mins (4°C). SYBRgold (Molecular Probes) was applied (1:10,000 in TE buffer, 10 mM Tris-Cl, pH7.5, 1 mM EDTA) to stain the DNA. Comets were imaged (Zeiss Axiovert 200 M, x10 lens) and measured using OPENCOMET free software 29 for Image J (v1.3).

### Isolation of breast cancer cells from primary samples and patient derived xenografts

Breast cancer cells were isolated from pleural effusions or ascites fluid from metastatic breast cancer patients (*n* = 4), using the following method. The acquired samples were centrifuged at 4°C for 10 min at 1000 × g, followed by resuspension in cold PBS. Red blood cells were removed using Lymphoprep™ (Axis-Shield) and subsequently leukocytes were removed with CD45-negative magnetic sorting (Miltenyi Biotech), according to the manufacturer's instructions. Metastatic human breast cancer samples were collected from patients at The Christie NHS Foundation Trust. All patients underwent fully informed consent, in accordance with local research ethics committee guidelines (05/Q1403/159 and 05/Q1402/25). The PDX model BB6RC50 was generated from the subcutaneous injection of 1 million metastatic breast cancer cells into NOD scid gamma mice. Passage 2 tumors were removed, dissected into 2–3-mm cubed pieces and then digested for 1–2 hours at 37°C in serum-free Dulbecco's modified Eagle Medium (DMEM;Gibco) containing × 1 collagenase/hyaluronidase (STEMCELL technologies) and penicillin (100 U/mL) – streptomycin (0.1 mg/mL; Sigma). The enzymatically digested tumor was then filtered through a sterile 40-μm mesh/sieve, to obtain a single-cell suspension and washed in cold PBS.

### Mammosphere culture of breast cancer cells

Mammospheres were cultured as described [30]. In brief, single cells from MCF7 and MDA MD 231 cell lines or primary metastatic samples were seeded at 500 cells per cm^2^ on poly-HEMA-coated plates. Mammosphere forming efficiency (MFE) was calculated as follows: [(number of mammospheres formed (≥60 μm) ÷ by the number of cells seeded) × 100].

### Tumor initiation and Extreme limiting dilution assay (ELDA)

MDA MB 231 cells were stained with MitoTracker and sorted into two populations; mito-low (lowest 5% mitochondrial mass) and mito-high (highest 5% mitochondrial mass). Cells were then resuspended in 1:1 solution of mammosphere media and growth factor reduced matrigel. NOD scid gamma (NSG) mice were then subcutaneously injected with 1, 5, 10 and 50 cells per flank (*n* = 3 mice per group) of mito-low and mito-high MDA MB 231 cells. Tumor size was measured bi-weekly (L × W × W/2) with calipers over 9 weeks (<100 μm = no tumor initiation). The tumor initiation rate from each population was then used to calculate tumor initiating cell frequency, using the extreme limiting dilution analysis calculation: http://bioinf.wehi.edu.au/software/elda/ [32].

### Statistical analysis

*In vitro* data is represented as the mean ± standard error of the mean (SEM), taken over ≥3 independent experiments, with ≥3 technical replicates per experiment, unless otherwise stated. Statistical significance was measured using the analysis of variance (ANOVA) test with post-hoc dunetts multiple comparisons or *t*-test, using Graphpad prism. *P* ≤ 0.05 was considered significant and all statistical tests were two-sided. All statistics were carried out under the guidance of the Medical Statistics Department, Christie Hospital NHS Trust, UK.

## Abbreviations

CSCs: cancer stem-like cells
TICs: tumor-initiating cells
PDX: patient-derived xenograft
ALDH: aldehyde dehydrogenase
